# MAPK/ERK Pathway as a Central Regulator in Vertebrate Organ Regeneration

**DOI:** 10.3390/ijms23031464

**Published:** 2022-01-27

**Authors:** Xiaomin Wen, Lindi Jiao, Hong Tan

**Affiliations:** Lab of Tissue Engineering, College of Life Sciences, Northwest University, Xi’an 710069, China; wenxmnwu@163.com (X.W.); jiaolindi_nwu@163.com (L.J.)

**Keywords:** MAPK/ERK pathway, organ regeneration, appendage regeneration, heart, liver, eye, nervous system

## Abstract

Damage to organs by trauma, infection, diseases, congenital defects, aging, and other injuries causes organ malfunction and is life-threatening under serious conditions. Some of the lower order vertebrates such as zebrafish, salamanders, and chicks possess superior organ regenerative capacity over mammals. The extracellular signal-regulated kinases 1 and 2 (ERK1/2), as key members of the mitogen-activated protein kinase (MAPK) family, are serine/threonine protein kinases that are phylogenetically conserved among vertebrate taxa. MAPK/ERK signaling is an irreplaceable player participating in diverse biological activities through phosphorylating a broad variety of substrates in the cytoplasm as well as inside the nucleus. Current evidence supports a central role of the MAPK/ERK pathway during organ regeneration processes. MAPK/ERK signaling is rapidly excited in response to injury stimuli and coordinates essential pro-regenerative cellular events including cell survival, cell fate turnover, migration, proliferation, growth, and transcriptional and translational activities. In this literature review, we recapitulated the multifaceted MAPK/ERK signaling regulations, its dynamic spatio-temporal activities, and the profound roles during multiple organ regeneration, including appendages, heart, liver, eye, and peripheral/central nervous system, illuminating the possibility of MAPK/ERK signaling as a critical mechanism underlying the vastly differential regenerative capacities among vertebrate species, as well as its potential applications in tissue engineering and regenerative medicine.

## 1. Introduction

Damage to organs by trauma, infection, diseases, congenital defects, aging, and other injuries causes organ malfunction and is life-threatening under serious conditions. One of the most exciting current biomedical research challenges is to decipher the molecular basis of organ regeneration, aiming to maintain, improve, and restore organ functions after injury. The regeneration capacity across vertebrate taxa varies greatly. Mammals, in particular humans, are not characterized as regeneration-competent species, with limited organ/tissue regenerative ability upon injury [1,2]. Lower order vertebrate animal phyla, such as fish, amphibians, and reptiles, in contrast, can withstand serious organ/tissue injury by efficient organ regeneration [2,3].

The phenomenon of organism regeneration has been documented dating back to the naturalist Aristotle [4]. However, due to a lack of laboratory tools and technologies, scientists have begun to address mechanistic questions on regeneration processes only in recent decades. Organ regeneration consists of a series of dynamic and complex processes that are exquisitely orchestrated by the interplay among extracellular and intracellular signaling, cytokines, growth factors, and other components [3]. Extensive studies have been carried out to unravel the molecular mechanisms that facilitate organ regeneration. Importantly, understanding of the molecular basis of organ regeneration holds the key to a new epoch for regenerative medicine for humans [5]. Currently, a growing number of studies underscore the significant and divergent roles of the mitogen-activated protein kinase (MAPK)/extracellular signal-regulated kinase (ERK) pathway (also known as the Ras/Raf/MEK/ERK pathway) in organ regenerative processes. However, the full spectrum of ERK signaling in organ regeneration at the cellular and mechanistic level has not been discussed. In this review, we recapitulate and discuss the advances of MAPK/ERK signaling regulations, its dynamic spatio-temporal activities, and the divergent roles contributing to organ regeneration, including appendages, heart, liver, eye, and central/peripheral nerve regeneration, in order to shed light on the development of tissue engineering and regenerative medicine.

## 2. MAPK/ERK Structure, Activation, and Function

**Figure 1:**
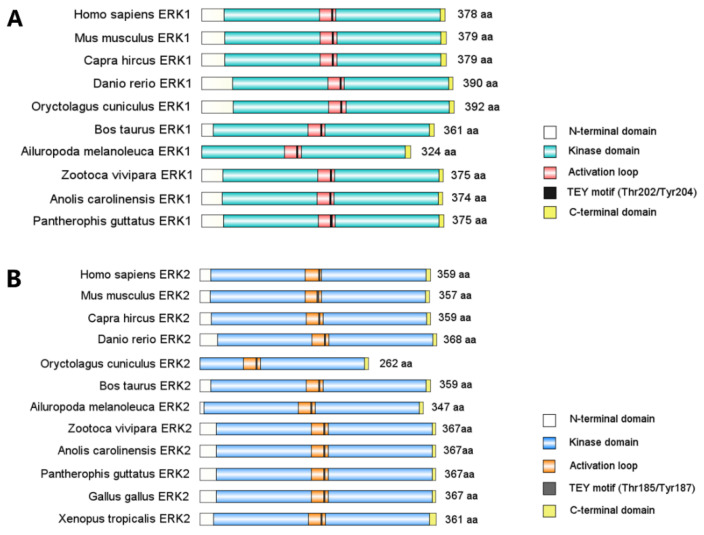
Diagram represents highly conserved ERK1/2 protein functional domains among vertebrates. Full length ERK1/2 amino acid sequences of vertebrate species were retrieved from the NCBI database (http://www.ncbi.nlm.nih.gov, accessed on 22 January 2022). ERK1/2 functional domains were mapped using IBS software and recolored. (**A**) ERK1 functional domain alignment of 10 vertebrate species. (**B**) ERK2 functional domain alignment of 12 vertebrate species.

The extracellular signal-regulated kinases, ERK1 and ERK2, are the prototype of ubiquitously expressed proline-directed Ser/Thr protein kinases that belong to the MAPK family. Only one ancestral form of ERK has been identified so far in invertebrates [6], while ERK1 and ERK2 protein sequences and structures maintain stable and highly homologues among vertebrate taxa (Figure 1). Moreover, ERK1 and ERK2 amino acid sequences are 84% identical across the mammal phylum [6]. Structurally, ERK1/2 both have the D-recruitment site and F-recruitment site as substrate binding domains [7] and a Thr-Glu-Tyr (TEY) motif in their activation segment for catalytic activation [8]. ERK1 and ERK2 are activated indiscriminately by the same extrinsic stimuli and share 22 out of 23 amino acids that directly phosphorylate their substrates [8,9,10]. A diverse array of extrinsic signals are transduced through MAPK/ERK signaling, including growth factors such as fibroblast growth factor 2 (FGF2) [11,12], platelet-derived growth factor (PDGF) [13,14], and insulin-like growth factor type 1 (IGF-1) [15]; neutrophins such as Neuregulin-1 [16], brain-derived neurotrophic factor (BDNF) [17], neurotrophin-3 (NT3) [18], and serotonin [19]; cytokines such as tumor necrosis factor (TNF) [20,21] and transforming growth factor (TGF)-α [22]; and cellular stress such as reactive oxygen species (ROS) [23], Ca^2+^ signaling [24], and DNA damage [25,26].

**Figure 2:**
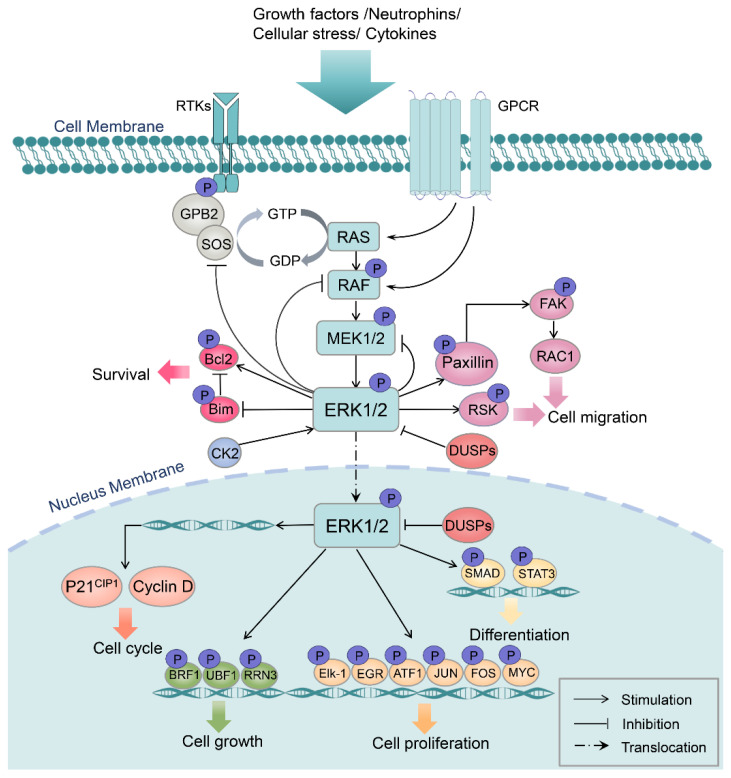
Simplified schematic of the regulatory mechanism and functions of the MAPK/ERK1/2 pathway. Upon receiving extracellular excitatory input, the Ras/Raf/MEK/ERK signaling cascade is activated and relayed through a three-tiered phosphorylation wave that occurs starting on the cell membrane. Activated ERK1/2 subsequently phosphorylate a broad range of substrates in the cell membrane, cytoskeleton, cytoplasm, and nucleus to execute essential cellular functions. GPB2, guanine nucleotide-binding protein subunit beta 2; SOS, son of sevenless; MEK, mitogen-activated protein kinase kinase; Bcl2, B cell lymphoma 2; CK2, casein kinase 2; FAK, focal adhesion kinase; RAC1, Ras-related C3 botulinum toxin substrate 1; RSK, ribosomal S6 kinase; DUSPs, dual-specificity phosphatases; BRF1, butyrate response factors 1; UBF1, upstream binding factor 1; EGR, early growth response; ATF1, activating transcription factor 1; STAT3, signal transducer and activator of transcription 3.

Activation of the Ras/Raf/MEK/ERK signaling cascade is relayed through hierarchical three-tiered phosphorylations that occur starting on the cell membrane. Once activated by the ligand–receptor interaction, Ras induces Raf dimerization and kinase activity, followed by the sequential phosphorylation of the serine residue on the dual-specificity kinases MEK1/2, which are highly exclusive in that ERK1/2 are their only known substrates. MEK1/2 continue to activate the downstream ERK1/2 (MAPK) by phosphorylation at Tyr 204/187 and Thr 202/185 sites. Then, activated ERK1/2 phosphorylates a broad variety of substrates localized in the cell membrane, cellular organelles, and cytoplasm. In addition, dimerized ERK can rapidly shuttle into the nucleus to regulate cell transcriptional activities through phosphorylating a number of transcription factor targets. Reciprocally, cytosol ERK1/2 can phosphorylate the upstream kinases of the ERK pathway, such as son of sevenless (SOS), Raf, and MEK, as a negative feedback regulatory mechanism (Figure 2) [27,28,29,30].

Functionally, since MAPK/ERK1/2 target over 600 substrates within the cell, they serve as a central hub that governs fundamental cellular behaviors including cell survival, differentiation, proliferation, growth, migration, and metabolism [31,32]. These cellular responses are critical for efficient organ regeneration, implying substantial involvement of the MAPK/ERK pathway in regeneration. Mounting research has evidenced a central role of MAPK/ERK1/2 signaling pathway in regulating complex organ regeneration.

## 3. Involvement of the MAPK/ERK Pathway in Tissue/Organ Regeneration Processes

To achieve successful organ regeneration, organ cells firstly sense injury-induced extrinsic signals, and subsequently, a set of intercellular and intracellular signaling is excited to execute regeneration processes in a meticulously coordinated manner including cell fate turnover, migration, proliferation, growth, and transcriptional and translational activities. By experimenting on regenerative/non-regenerative vertebrate animal models (*Xenopus*, zebrafish, axolotl, newt, chick, mice, and rat), tremendous effort has been put in to investigate molecular mechanisms regulating tissue/organ regeneration processes and determined that the MAPK/ERK pathway plays diverse and profound roles in regulating an array of tissue/organ regeneration, as briefly summarized in Table 1.

### 3.1. MAPK/ERK Pathway in Appendage Regeneration

Appendage regeneration mostly occurs in lower order vertebrates such as teleost fish and urodele amphibians. It represents a typical epimorphic regeneration model that injured or lost appendages can be fully reconstituted both anatomically and functionally [83]. In contrast, injured mammal extremities go through pathological healing processes resulting in excessive inflammation and scar formation [1]. Precise orchestrations among a complex variety of cell and tissue types are required during appendage regeneration. Take axolotl limb regeneration, for example: upon injury, surrounding epidermal cells are rapidly activated and migrate, forming the wound epithelium over the wound surface within 24 hr. Then, local terminally differentiated cells including dermal cells, epidermal cells, skeletal muscle cells, Schwann cells (SC), chondrocytes, and fibroblasts, among others, become motivated and go through dedifferentiation to turn into progenitor cells, which interestingly, are lineage-restricted progenitors with different levels of plasticity [84,85,86]. As a result, these heterogeneous progenitor cells accumulate underneath the thickened wound epithelium, forming a cone-shaped stump called blastema, and continue to outgrow into a replica of the lost body part [2].

MAPK/ERK signaling is determined to play divergent and beneficial roles in appendage regeneration. MAPK/ERK activation as one of the early cellular responses has been described across species, implicating its conserved role in the initiation of regeneration [36,37,38,39,40]. The first cellular responses triggered by injury are pivotal for regeneration onset, such as ROS production and Ca^2+^ signaling activation [87,88,89]. Multiple studies demonstrate ERK activity directly links to effects of first cellular responses. Sato et al. [37] showed that early ERK activation induces ROS production via promoting TGF-β signaling to initiate regeneration. Inhibition of the ERK/TGF-β/ROS signaling cascade by treatment with chemical inhibitors for ERK, TGF-β, or ROS sabotage stump epidermis formation and wound closure in *Xenopus laevis* tail regeneration. In addition, Ca^2+^ signaling, another immediate wound signal, is evoked rapidly post appendage injury in *Xenopus* [90], zebrafish [91], and axolotl [92], and plays significant roles in regeneration initiation. Ca^2+^ signaling is known to trigger rapid ERK activation post injury to promote regeneration [93].A study shows that inhibition of the sarco/endoplasmic reticulum Ca^2+^ ATPase (SERCA) at low concentrations blocks axolotl tail regeneration completely [36]. In addition, blockade of the Ca^2+^ activated chloride channel, anoctamin1, results in reduction of cell proliferation and significantly diminishes the MAPK/ERK pathway activation [36]. Overall, MAPK/ERK signaling is promptly excited upon injury stimuli and integrates with other immediate early response signaling to promote regeneration initiation.

Dedifferentiation of local mature cells is a hallmark of blastema formation. Multiple studies demonstrate suppression of p53 as a key mechanism to allow cell cycle re-entry during cell dedifferentiation [94,95]. A comparative study by Yun and colleagues [33] investigating myotube dedifferentiation highlights suppression of p53 activity by sustained ERK activation (over 48 h) during salamander myotube dedifferentiation and proliferation, which is distinctive from the transient ERK activation pattern (less than 3 h) and failure of dedifferentiation induction in mammal myotubes. Moreover, sustained ERK activation alters epigenetic modification of H3K9 demethylation and down-regulates muscle-specific gene expressions. These findings suggest that firstly, insufficient ERK activation is at least partly obligatory for the weak myocyte regenerative ability in mammals. Secondly, sustained ERK activation functions via diverse routes to achieve salamander myotube cell cycle progression and cell fate turnover. However, inhibition of FGF/VEGF, the classic growth factors to elicit ERK activation, shows no inhibitory effect on the sustained ERK activation pattern in salamander myotubes, while another study in zebrafish [96] proves skeletal muscle dedifferentiation is dependent on FGF/ERK signaling activation. The irresponsiveness of ERK activity to FGF/VEGF inhibition in salamander myotubes infers the likelihood of novel molecules to ignite and maintain the special ERK activation pattern, or the unique specificities of the RTK receptor.

Importantly, ERK signaling functions beyond intracellularly in that it also has significant impacts intercellularly on tissue morphogenesis. Genetic modifications targeting the ERK pathway have greatly facilitated understanding the dynamic flow of ERK activity in vivo. Recent work by De Simone et al. [41] determined ERK plays a profound role in choreographing growth and patterning of fish scale regeneration. By constructing Erk kinase translocation reporter (Erk KTR) transgenic zebrafish to track cellular ERK activity level, results show ERK activity follows a delicate temporal-spatial pattern during zebrafish scale regeneration, which expands from the center across the whole scale, forming continuous concentric ring waves. Perturbation of ERK signaling waves causes disruptive growth pattern and defective scale morphogenesis in size and shape. Concomitant with this study, findings from Hino et al. [97] elucidated mechanisms of ERK signaling mediating the mechanochemical-induced collective cell migration in vitro. In a light-inducible ERK activation system, live imaging shows ERK activation waves propagated from leader cells to follower cells. Induction of the EGFR/ERK/Rho-associated kinase signaling cascade causes cell front-rear polarization and cytoskeleton contraction to relay intercellular contraction forces and thereby orchestrate unidirectional collective cell migration. These findings suggest that the ERK pathway acts as one of the master pathways in orchestrating overall tissue growth and patterning for proper regeneration.

Intriguingly, the MAPK/ERK pathway is shown to participate in the rare case of post-natal mammal appendage regeneration, such as in deer antler regeneration. It is classified as epimorphic regeneration that occurs periodically without injury stimuli [98]. Proteomics analysis of regenerating red deer antler indicates MAPK/ERK signaling is activated in both pedicle periosteum and antlerogenic periosteum cells, which are the major cell sources to form the antler bud [44]. By investigating a component protein purified from deer antler tissue named pilose antler peptide (PAP), Yun et al. [43] elucidates how the administration of PAP strongly activates insulin signaling and hierarchically stimulates ERK and PI3K/Akt signaling, to promote osteoblasts proliferation, differentiation, and mineralization.

Of note, by using a bioinformatics screen among cold- and warm-blooded vertebrate animal genomes, a new gene named cold-blooded animal-specific wound epithelial receptor-binding gene (*c-answer*) was identified, which is homologous to FGFRs and plays critical roles in *Xenopus laevis* hindlimb/tail regeneration [99]. Mechanistic studies performed on the animal cap of *Xenopus* embryo reveal that C-answer homodimer forms a complex with FGF8 and FGFR1-4 on the cell membrane that significantly stimulates MAPK/ERK signaling. Another novel protein discovered by Brockes lab is a salamander-specific neurotrophic protein named newt anterior gradient protein (nAG), which induced nAG over-expression at the amputation site and is sufficient to rescue the regeneration ability of the denervated newt limb [100]. Separate studies from the same lab identified prod1 as a blastema cell surface receptor for nAG [101], and EGFR/ERK1/2 signaling is robustly activated in Prod1 over-expressed newt blastema cells, leading to a soaring increase of MMP9 transcription and expression [34]. High MMP9 activity is important for wound epithelium/blastema formation owing to its function in regeneration-related ECM remodeling [102,103]. From these studies, we deduce that ERK activation is required in mediating pro-regenerative effects of some novel proteins discovered in regeneration-competent vertebrate animals. Nevertheless, to draw confirmative conclusions, explicit evidence to link ERK signaling to these novel proteins in the context of appendage regeneration is in demand.

### 3.2. MAPK/ERK Pathway in Cardiac Regeneration

The mammal heart is considered to be a non-regenerative organ, due to the permanent postmitotic arrest of cardiomyocytes (CMs), whereas zebrafish [104,105,106], amphibian [107], and neonatal mouse heart [108] can efficiently regenerate through robust CMs refuel from pre-existing CMs and neovascularization contributed by epicardial and endocardial cells. After injury, the surrounding epicardial cells migrate to cover the wound, followed by dedifferentiation of CMs, which features sarcomere disassembly, reduced CM marker genes expression, and cell cycle re-entry. Then, with robust ECM rearrangement and cytoskeletal remodeling, the proliferating CMs go through an epithelial–to-mesenchymal transition (EMT)-like process to integrate into and replace the damaged myocardium [109,110].

MAPK/ERK signaling exhibits beneficial and multifaceted roles in inducing CMs reprogramming and neovascularization following heart resection or infarction. ERK signaling integrates in the dynamic flow of signal networks, and each individual pathway is engaged in designated cellular modifications. Among these signaling pathways, the ERK/Yes-associated protein (YAP) axis is prominent in reawakening CM cell plasticity [47,49]. Multiple studies [48,52] underline the essential role of ERK signaling in mediating the potent effects of Erb-B2 Receptor Tyrosine Kinase 2 (ERBB2) activation in CM, which facilitates postnatal mice CM dedifferentiation and proliferation, as well as the subsequent redifferentiation and regeneration. A more recent study also reveals transient overexpression of activated ERBB2 in CMs stimulates ERK signaling [47]. Activated ERK hierarchically induces YAP activation to drive myoskeleton and nuclear-envelope components alteration, which causes sarcomere disassembly, EMT behavior, and robust CMs proliferation. Excitation of ERBB2/ERK/YAP signaling is sufficient to reactivate juvenile and adult mice CMs’ regenerative potential. Likewise, agrin, a neonatal mice ECM glycoprotein, through binding to the receptor α-Dystroglycan (Dag1), activates the downstream ERK and YAP to efficiently promote CMs’ dedifferentiation, maturation, and proliferation in myocardium infarction (MI) adult mice [49]. To make a clue of how ERK carries out its divergent cellular functions, it is critical to identify its key substrates that execute specific cellular changes in different contexts. Here in cardiac regeneration, strong YAP nuclear translocation and activation to promote myoskeleton rearrangement under the control of ERK signaling are confirmed to be pivotal.

Of importance, increased ERK activity during cardiac regeneration is suggested to guide coronary vasculature, which is vital for supporting and nurturing regenerated heart tissue. Elevated MAPK/ERK pathway activation is induced in the epicardium, endothelium, and injury border zone in zebrafish heart upon injury [45,111]. Robust H_2_O_2_ (~30 μM) is released immediately at the site post-injury, and it subsequently targets and destabilizes a potent pERK dephosphotase called dual-specificity phosphatase 6 (Dusp6) [45]. Suppression of Dusp6 unleashes ERK1/2 activities to promote angiogenesis and CMs proliferation, as well as to reduce fibrosis after partial resection in zebrafish heart [45,46]. Moreover, proper coronary vasculature morphogenesis and neovasculature stabilization require tight control of cell–cell adhesion among homotypic and heterotypic cells, for instance, endothelial cells and CMs. In the MI rat model, over-expression of N-cadherin significantly increases ERK activity, which in turn promotes vascular endothelial growth factor (VEGF) expression [50]. The up-regulated VEGF functions in a paracrine manner contributing to neovascularization and integration of regenerated CMs.

Interestingly, epigenetic modification on ERKs by the long non-coding RNA (lncRNA) plays a significant role in cardiac regeneration. A highly up-regulated novel lncRNA named endogenous cardiac regeneration-associated regulator (ECRAR) was identified by an unbiased screen of lncRNA transcriptome of fetal and adult human heart [53]. Using the cardiac Ad-ECRAR transfection approach, Chen et al. [53] showed that ECRAR physically binds to ERK1/2 in the cytoplasm. After activation, ERK1/2 translocate into the nucleus and increase expression of pivotal cell cycle control molecules including cyclin D1/E1 and E2F1, thereby forming a E2F1-ECRAR-ERK1/2 positive feedback signaling loop to reactivate CMs proliferation and regeneration after MI. This study provides an insight that even though ERK1/2 ubiquitously exist in all cell types and are highly conservative, differential epigenetic modifications among various species are one of the regulatory mechanisms to individualize ERK activities such as the subcellular localization, and thereby to switch cell fate in diverse directions.

### 3.3. MAPK/ERK Pathway in Liver Regeneration

The liver is a unique inner organ from the perspective that even the vertebrate taxa conserve high liver regeneration capacity [112]. The liver is capable of regenerating up to 70% of liver mass, in which residue hepatocytes make the most contribution through proliferation and differentiation [113]. After partial hepatectomy (PH), the remaining hepatocytes re-enter the cell cycle within 12 h, followed by proliferation of other cell types such as cholangiocytes and Kupffer cells. Then, through a remodeling phase, normal liver structure and functions are restored [114,115].

Endocrine hormones and growth factors are potent hepatic mitogens, through which the HGF/MET [116], insulin-like growth factor type 1 (IGF-1)/IGF-R [56], and growth hormone (GH)/GHR [60] pathways are among those well established for efficient liver regeneration. Several studies support ERK as one of the underlying core signaling pathways that promote hepatocyte and cholangiocyte proliferation during liver regeneration [54,55,56,57,60,61,117]. Liver regeneration is severely impaired in GHR knockout mice and shows suppressed EGFR expression and blocked EGFR phosphorylation [60]. Further investigation identified ERK1/2 as the downstream mediator of EGFR signaling in promoting hepatocyte G_1_ to S phase cell cycle progression. Another study [56] on the effects of IGF-1 utilizing the liver-specific IGF-1R knockout mice demonstrates that although IGF-1R function is not required in maintaining a healthy liver, the IGF-1R/IRS-1/ERK signaling axis is a requisite to induce hepatocyte proliferation after liver resection. By using the L-O2 cell line and serotonin-deficient transgenic mice, Yu et al. [55] discovered that serotonin treatment significantly increases the expression and activity of YAP via ERK signaling, which induces hepatocyte proliferation and liver function restoration during regeneration.

Excitingly, understanding of the critical role of the ERK pathway has been applied in liver tissue engineering construction [58,59]. A recent work reported generation of tissue-engineered liver organoids with a small-molecule cocktail by targeting the PKA/ERK, Wnt/β-catenin, and NMII/Rac signaling pathways, respectively. Modulation of this signaling set induced mouse liver organoids expansion as well as achieved long-term (>20 passages) ex vivo maintenance [58]. Kim et al. [59] developed a small-molecule cocktail composed of HGF, A83-01, and CHIR99021, which activates MET/ERK signaling and suppresses TGF-β and GSK3 signaling, respectively. By administration of this cocktail, human hepatocytes isolated from healthy/diseased donor livers are reprogrammed into bipotential human hepatic progenitors that can differentiate into hepatocytes or cholangiocytes. Moreover, this population of hepatic progenitors is able to repopulate in injured liver and restore liver functions after being intrasplenic transplanted in mice models. Therefore, development of pharmacological activation of the MAPK/ERK pathway could be a promising therapeutic strategy to benefit patients with liver injury.

### 3.4. MAPK/ERK Pathway in Eye Regeneration

Mammals are susceptible to irreparable degenerative retinal diseases due to their defective eye regeneration capacity [118]. In contrast, amphibians, teleost fish, and avians are able to fully regenerate their damaged retinas [62,63,64,65,66,67,68,69]. Retinal pigmented epithelium cells (RPE) [67,69,119] in newt, *Xenopus*, and embryonic chicks, and Müller glia cells (MG) [62,63,120,121] in teleost fish, *Xenopus*, and post-hatched chicks are the major cell sources for retina regeneration through transdifferentiation and proliferation. During transdifferentiaton, quiescent RPE/MG become stimulated to dedifferentiate and proliferate into multipotent neuroepithelial cells, which continue to differentiate into all cell types required to rebuild the retina [62,122,123].

Besides the striking similarity of regeneration strategy employed to regenerate a retina, several studies indicate that ERK is also employed as a key mechanism promoting retina regeneration across species. Through years of work, Chiba’s laboratory [64,65,66,67] posits a multi-step mechanism regulating retinal regeneration in the adult newt. Firstly, strong immediate early activation of ERK signaling as well as prominent p-ERK nuclear translocation take place in retinal RPE within 30 min following retinectomy. Next, the early activated ERK signaling and loosened cell–cell contact cause nuclear translocation of β-catenin at around 3 days post injury. Subsequently, extracellular factors such as FGF2 induce a reinforcement of ERK activation that continues to act synergistically with β-catenin signaling and other heparin-binding (HB) signaling to promote cell cycle re-entry, transdifferentiation, and proliferation of RPEs. By performing the surgical procedure to implant heparin-coated FGF2 beads inside the optic cup of chicks [69] and *Xenopus laevis* [68] after retinectomy, studies show exogenous FGF2 works through ERK signaling to increase paired box 6 (*pax6*) expression, a key transcription factor controlling RPE reprogramming.

In line with the above findings, during zebrafish retina regeneration, ERK signaling in MG cells becomes activated upon stimulation by multiple growth factors (HB-EGF, FGF2, IGF1, and insulin) [62,63]. It then exerts functions collectively with other signaling (β-catenin, pStat3) in cytoplasm and, simultaneously, functions in the nucleus to induce transdifferentiation-related transcription factor expressions (*pax6_b_*, *ascl1a*), thereby promoting transdifferentiation and proliferation of quiescent MG [62,63]. To sum up, these findings indicate ERK signaling mediates similar cellular responses in RPE and MG and yields equivalent outcomes in retina regeneration across phyla.

### 3.5. MAPK/ERK Pathway in Central/Peripheral Nerve Regeneration

The central nervous system (CNS) consists of the brain and the spinal cord, whereas the peripheral nervous system (PNS) includes the rest of the nerve networks bridging the CNS and tissue/organs [124]. Vertebrates can recover well from PNS injury, during which Schwann cells (SCs), the peripheral nerve glia cells, take a leading role in orchestrating peripheral nerve regeneration [125]. SCs exhibit remarkable plasticity during nerve regeneration. SCs initially undergo demyelination and dedifferentiation, along with recruited immune cells, to facilitate Wallerian degeneration, followed by proliferation and differentiation to form the bands of Büngner to re-establish axon connection and further guide axon regeneration [126,127]. Acute strong pERK expression in SCs is induced in sciatic-nerve-transected animals [71]. By using transgenic approaches to construct inducible Raf SCs and transgenic mice, solid data from Lloyd lab [71,72] indicates the following: (1) single activation of Ras/Raf/ERK pathway in SCs is sufficient to induce SCs dedifferentiation; (2) continual Ras/Raf/ERK activation maintains SCs in the dedifferentiated state while suppressing SCs differentiation; and (3) Raf activation in SCs recruits immune cells on site, including macrophages, mast cells, neutrophils, and T cells. In addition, in a nerve tissue engineering study using a sciatic-nerve-transected rat model, SCs overexpressed with VEGF-A stimulate drastic elevation of VEGF/VEGFR2/ERK signaling, which is shown to promote neurological recovery, in particular angiogenesis [74]. Furthermore, the intercellular communications are also of great interest to scientists. Negro et al. [128] revealed degenerating neurons control SCs’ behavior by releasing ATP, which enhances ERK 1/2 and the cAMP-response element binding protein (CREB) phosphorylation, triggers cAMP production, and causes cytosol Ca^2+^ surge inside SCs. However, a study from Cervellini et al. [129] shows long-term ERK activation is detrimental to PNS regeneration. By constructing gain-of-function *MEK1DD* transgenic mice to induce constitutive ERK activation (over 6 weeks), results show impaired nerve regeneration in *MEK1DD* transgenic mice after sciatic nerve transaction, which is due to suppressed SCs’ differentiation and axonal remyelination from long-term ERK activation. Overall, the contradictive regeneration outcomes [72,129] deriving from suppressed SC differentiation by ERK activity underscore the significance of precise control of SC plasticity alterations during PNS regeneration.

Nevertheless, few PNS regions in mammals have lost regeneration ability during evolution, for instance the cochlear and vestibular hair cells in the auditory and vestibular systems. Loss of hair cells leads to hearing loss and balance disturbance in mammals, whilst hair cells in non-mammal vertebrates (fish, birds, reptiles, and amphibians) maintain homeostasis through active turnover [130,131]. In investigating zebrafish hair cell regeneration, Bao et al. [70] demonstrated histone H3 at lysine 27 (H3K27) demethylase is critical in promoting regeneration of hair cell and neuromasts via ERK-dependent cell cycle progression. This study indicated that the H3K27 epigenetic modification on ERK signaling may be one of the mechanisms underlying differential hair cell regeneration capacities among species.

In contrast to PNS regeneration, injuries to CNS in mammals are catastrophic, largely due to failure of axon regrowth, whereas fish [132,133], frogs [133,134], lizards [135], and salamanders [133,136,137] are able to recover from CNS loss. Oligodendrocytes (OLs) are the major glial cell type to form myelin ensheathing axons in CNS, which are differentiated from oligodendrocyte progenitor cells (OPCs) during regeneration. Numerous studies evidence the essential roles of the MAPK/ERK pathway in CNS development and regeneration. To systemically investigate roles of the MAPK/ERK pathway in nervous system (CNS and PNS) development, Ishii and colleagues [73,78] conducted loss-of-function and gain-of-function studies by generating glia-specific ERK1/2 double knock-out and constitutive active ERK1/2 transgenic mice, respectively. Both in vivo and ex vivo experiments prove that enhanced MAPK/ERK activities increase OLs/SCs myelin sheath thickness and OPCs proliferation in the cerebellum, brainstem, spinal cord, and sciatic nerve development. In addition, during CNS regeneration, the MAPK/ERK pathway is under multifaceted control and exerts diverse functions. In a study investigating optic nerve regeneration in frogs, retinoic acid is shown to maintain long-term survival of retinal ganglion via activating MAPK/ERK and STAT3 signaling [75]. In rodent CNS injury models, the ERK/CREB pathway is evidenced to be stimulated by signal molecules such as BDNF [80], anti-apoptotic protein Bcl-2 [24], and intracellular sigma peptide (ISP) [81] to promote neuronal survival, neurite outgrowth, and axon remyelination, thereby facilitating CNS regenerative processes. By the transgenic manipulation approach, sortilin-related receptor with A-type repeats (SORLA), a transmembrane trafficking protein expressed by neurons, is shown to promote neurite outgrowth and regeneration through elevating the EGF receptor/ERK/c-fos axis [76]. During severe facial nerve axotomy regeneration, the MAPK/ERK and PI3K/Akt pathways enhance axon regrowth and facial nucleus neuron survival, separately, indicating the importance of inter-signaling collaborations [82]. Excitingly, miconazole, a Food and Drug Administration (FDA)-approved blood–brain-barrier-crossing drug, is currently under repurposed drug development to promote OPCs differentiating into OLs, as well as OLs remyelination for multiple sclerosis treatment, which functions through activating MAPK/ERK signaling [77]. Nonetheless, several recent studies describe ERK activation as detrimental to central nerve regeneration. In a drug-induced demyelination mice model, administration of ERK inhibitor promotes OPCs differentiation and myelin formation in spinal cord and corpus callosum regeneration [138]. In another study by Xue and colleagues, using a tripartite motif containing 32 (TRIM32)-lentivirus infecting neural stem cells (NSC) at spinal cord injury (SCI) site in mice, the results show that blockade of the EGFR/ERK pathway upregulates TRIM32, a neuronal differentiation factor, promoting NSCs’ differentiation into mature neurons, as well as functional recovery post SCI [79].

According to the above studies, MAPK/ERK activation yields mixed results in CNS and PNS regeneration, which promotes neuronal survival, SC dedifferentiation, glial progenitor cell proliferation, axon outgrowth, and angiogenesis, but suppresses SC/NSC differentiation and the subsequent remyelination. The contradictory effects of MAPK/ERK signaling can be partly explained by its stage-specific and cell-type-specific attributes, which, when designed in different experimental settings, may give rise to conflicting regenerative outcomes. Nevertheless, its indispensable role in promoting nerve regeneration in the CNS and PNS should not be undermined. Due to the complex glial/neural plasticity alterations during nerve regeneration, precise control of spatio-temporal ERK activation and tight collaborations with other pathways in neurons and glial cells are requisites for successful nerve regeneration.

## 4. Conclusions and Perspectives

**Figure 3:**
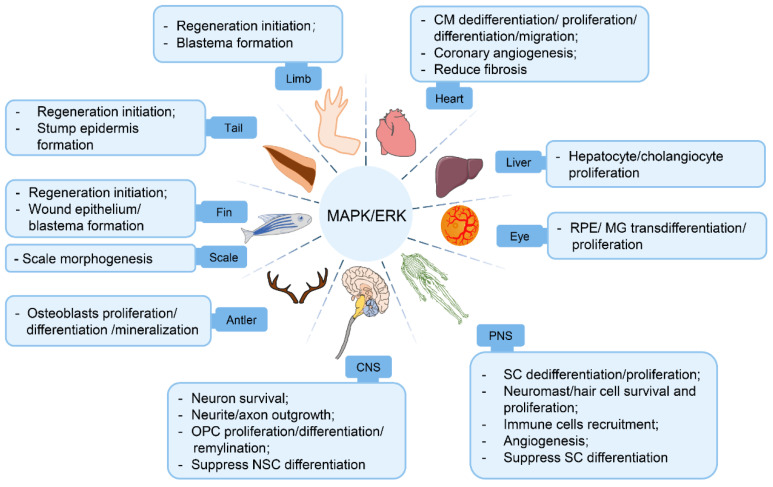
The role of MAPK/ERK signaling in vertebrate organ regeneration. Schematic of effects of MAPK/ERK activation on cellular responses and regenerative outcomes during multiple organ regeneration processes (appendages, heart, liver, eye, peripheral/central nervous system) in vertebrate animal studies.

Accumulating evidence to date has established a central role of the MAPK/ERK pathway in vertebrate organ regeneration, as it actively participates in regeneration initiation, tissue growth, morphogenesis, angiogenesis, and so on (Figure 3). The pro-regenerative effects of the MAPK/ERK pathway in organ regeneration are based on its functions in (1) facilitating an array of pro-regenerative cellular processes, (2) orchestrating homotypic/heterotypic intercellular communications, and (3) promoting ECM remodeling to create a regeneration-friendly microenvironment.

Importantly, it is worth pointing out the commonalities and discrepancies of ERK activities during different organ regeneration processes. Rapid ERK activation as an “alarm” signal upon injury is conserved at the onset of multiple organ regeneration. On the other hand, although MAPK/ERK exists ubiquitously in all cells, it is not surprising that the spatio-temporal dynamics of ERK signaling is customized in each organ due to their diverse cell/tissue composition and fluctuates dynamically throughout regeneration processes. As discussed above, sustained ERK activation is observed in salamander myotube turnover [33]; continuous reaction-diffusion trigger waves of ERK activities are evident throughout zebrafish scale regeneration [41]; and a reinforcement of ERK activation is detected following the retinectomy-induced immediate early ERK activation in newt retina regeneration [65]. These particular activation patterns are crucial because short bursts of ERK activation failed to induce mammal myotube regeneration [33], and perturbation of ERK activity waves sabotages proper morphogenesis in zebrafish scale regeneration [41]. In particular, continual or untimely ERK activation impairs neuron maturation and axonal myelination in both CNS and PNS regeneration [79,129]. MAPK/ERK signaling activities are under the influence of complex regulatory factors as discussed above, such as ligand–receptor interactions, subcellular compartments localizations, epigenetic modifications, and so on. Keyes et al. [139] conducted experiments in PC12 cells showing that EGF induces sustained ERK activation and causes cell morphology change when located near the plasma membrane, compared to a transient ERK activation when located in the cytoplasm and nucleus. It is fascinating how ERK signaling regulation is under such meticulous control. Therefore, it is necessary to rigorously test the distinctive ERK activation patterns (timing, strength, duration, and cell type) in different organs to fully understand the effects and mechanisms of the ERK pathway in each organ regeneration scenario.

Despite our emphasis on MAPK/ERK signaling in organ regeneration, the regulation is not a linear process by nature; rather, several signaling pathways actively intertwine with each other. At the onset of regeneration, organ cells receive injury-induced extracellular stimuli that can ignite a variety of intracellular signaling pathways simultaneously. Nonetheless, these pathways act on all kinds of cellular machines that may lead the cellular modifications into conflicting directions. Therefore, to achieve successful regeneration, especially on a whole organ level, it is pivotal to build up a highly organized signaling network to guide all types of cells through specific transformations sequentially. During regeneration, the MAPK/ERK pathway undergoes delicate regulations/modifications, becomes turned on in unique spatio-temporal patterns, and interfaces with other pathways to act synergistically or in parallel. For instance, direct interactions among a core set of signaling pathways (MAPK/ERK, PI3K/Akt, β-catenin, and Jak/Stat signaling) are elucidated to collectively drive MG transdifferentiation and proliferation in zebrafish eye regeneration [63]. In initiation of zebrafish fin regeneration, FGF/ERK1/2 and Wnt/β-catenin signaling concomitantly regulate raldh2 expression, the retinoic acid synthesis enzyme, to promote wound epithelium and blastema formation [39,40]. Since Ras can activate both the Raf/MEK/ERK and the PI3K/Akt pathways [31], they develop close interactions during regeneration. In the context of *Xenopus* froglet limb and deer antler regeneration, the MAPK/ERK and PI3K/Akt pathways are excited simultaneously and collaboratively to drive cell cycle re-entry and dedifferentiation as well as suppress apoptosis [35,43], while to achieve peripheral nerve regeneration, they function in parallel in separate aspects [82]. In more complex contexts, such as CNS regeneration, ERK activity needs to be transiently suppressed to allow other signaling pathways to set in to induce neuron/glial progenitor cell differentiation. On the contrary, certain signals must be downplayed. For example, Dusp6 activity needs to be suppressed to increase ERK activity as manifested in cardiac regeneration [46]. In addition, Rb protein is inactivated through sustained ERK phosphorylating activity in order to permit myocytes to re-enter the cell cycle [33]. According to these studies, the presented multitasking roles of the MAPK/ERK pathway are at least partly due to its functional interactions with partner signaling pathways. More importantly, understanding of core signaling interactions has great potential in regenerative medicine development. For instance, multiple groups have successfully designed small-molecule cocktails to generate hepatic progenitors by targeting core sets of signaling pathways, including MAPK/ERK signaling [58,59]. However, the knowledge is far from complete regarding the dynamic crosstalk and on/off switch among MAPK/ERK and other signaling pathways, as well as their convergence on cellular machines during different types of organ regeneration. Thus, more in-depth investigations of the signaling network are required to develop an integrated picture of the regeneration system of each organ.

An interesting further question concerns the differentially distributed regeneration ability within evolutionarily closely related vertebrate phyla. Increasing studies have been carried out aiming to unravel the molecular and signaling basis of the discriminated regenerative capacity across species. One of the theories of defective regeneration ability in mammals is due to insufficient/missing/dysregulation of key signaling activation in mammal cells. Through unbiased transcriptomic screening and comparative studies across species, novel genes are identified, for instance, *c-answer* [99], *Agr* genes [100,140], *prod1* [101], and *Ras-dva1/2* [141], which are lost or modified during evolution but are still carrying their regenerative functions in lower order species. Among these molecules, c-answer and prod1 proteins have been revealed to evoke drastic MAPK/ERK signaling activation. However, there is still a long road ahead to comprehend the regeneration-involved molecules/signaling that are abandoned or conserved during evolution. Given the rapidly evolving toolbox in genetics and molecular biology, for instance, single-cell transcriptomics [142,143], transcription activator-like effector nucleases (TALEN) [46], and clustered regularly interspaced short palindromic repeats (CRISPR)/Cas9 [144] gene editing approaches, performing comparative studies among different cell types, organs, and species on a mass scale to pin down key pathways, epigenetic factors, and cell origins during organ regeneration [145] are becoming increasingly promising.

## Figures and Tables

**Table 1:** Overview of MAPK/ERK pathway in vertebrate organ regeneration.

Organs	(Species)	Signaling Components	Functions	References
Limb	(newt)	‑ ERK/p53/sox6	‑ Promote cell cycle progression and dedifferentiation	[33]
	(newt)	‑ Prod1/EGFR/ERK/MMP9	‑ Promote WE and blastema formation	[34]
	(*Xenopus laevis*)	‑ MAPK/ERK1/2 and PI3K/Akt pathways	‑ Promote blastema formation	[35]
Tail	(axolotl)	‑ anoctamin1/ERK1/2	‑ Promote cell proliferation	[36]
	(*Xenopus laevis*)	‑ ERK/TGF-β/ROS	‑ Promote wound healing	[37]
	(zebrafish)	‑ ERK	‑ Promote regeneration	[38]
Fin	(zebrafish)	‑ FGF/ERK1/2&Wnt/β-catenin/raldh2	‑ Promote WE and blastema formation	[39]
		‑ FGF/ERK1/2&Wnt/β-catenin	‑ Promote cell proliferation	[40]
			‑ Promote regeneration initiation	
Scale	(zebrafish)	‑ ERK activity wave	‑ Control scale morphogenesis	[41]
		‑ Melatonin/ inhibiting ERK1/2	‑ Suppress osteoblast and osteoclast differentiation	[42]
Antler	(deer)	‑ PAP/InsR/IRS-1/ERK and PAP/PI3K/Akt	‑ Promote osteoblasts proliferation, differentiation, and mineralization	[43]
		‑ ERK/MAPK, PI3K/Akt and p38 MAPK		[44]
Heart	(zebrafish)	‑ Duox&Nox2/H_2_O_2_/Dusp6/ERK1/2	‑ Promote CM proliferation, coronary angiogenesis	[45]
		‑ Dusp6/Ras/MAPK	‑ Reduce fibrosis	[46]
	(mice)	‑ ERBB2/ERK/YAP	‑ Reactivate adult mice CM dedifferentiation, proliferation, and migration	[47]
		‑ ERBB2/ERK	‑ Promote CM dedifferentiation, proliferation, and redifferentiation	[48]
		‑ Agrin/ Dag1 /ERK&YAP	‑ Induce adult/juvenile mice CM dedifferentiation, maturation, and proliferation	[49]
		‑ N-Cadherin/ERK/VEGF	‑ Promote coronary angiogenesis and CM integration	[50]
		‑ LPA/LPA_3_/ERK	‑ Promote CM proliferation	[51]
	(rat)	‑ ERBB2/ERK	‑ Induce CM proliferation, sarcomere loss, and tissue remodeling	[52]
		‑ E2F1/ECRAR/ERK1/2 positive feedback loop	‑ Promote adult CM proliferation	[53]
Liver	(axolotl)	‑ ERK activation	‑ Promote hepatocyte proliferation	[54]
	(mice)	‑ Serotonin/ERK/YAP	‑ Promote hepatocyte proliferation	[55]
		‑ IGF-1R/IRS-1/ERK/cyclin D1&A	‑ Promote hepatocyte proliferation	[56,57]
		‑ PKA/ERK, Wnt/β-catenin and NMII-Rac signaling	‑ Promote liver organoid cholangiocyte-to-hepatocyte differentiation, Expansion, and ex vivo maintenance	[58]
		‑ HGF/MET/ERK1/2, inhibition of TGF-β and GSK3 signaling	‑ Promote human hepatocytes cell fate turnover and proliferation	[59]
		‑ GHR/EGFR/ERK1/2	‑ Promote hepatocytes cell G_1_/S phase transition	[60]
	(rat)	‑ ERK1/2 & p70^S6K^	‑ Promote hepatocyte and cholangiocyte proliferation	[61]
Eye	(zebrafish)	‑ HB-EGF/EGFR/ERK1/2/a*scl1a*, *c-myc_b_*&*pax6_b_*	‑ Promote MG dedifferentiation	[62]
		‑ Insulin& HB-EGF, IGF-1& FGF2/ERK, PI3K/β-catenin and pStat3	‑ Promote MG reprogramming and proliferation	[63]

	(newt)	‑ MEK1/2/ERK1/2	‑ Promote RPE cell cycle re-entry	[64,65,66]
		‑ FGF2/MEK-ERK	‑ Promote RPE transdifferentiation and proliferation	[67]
	(*Xenopus laevis*)	‑ FGF2/MEK/ERK	‑ Promote RPE transdifferentiation and proliferation	[68]
	(chick)	‑ FGF2/FGFR/MEK/ERK/Pax6	‑ Promote RPE transdifferentiation	[69]
PNS	(zebrafish)	‑ H3K27me3 histone demethylase/ ERK1/2/*p21*&*p27*	‑ Promote hair cell proliferation	[70]
			‑ Promote neuromast proliferation and survival	
	(mice)	‑ Ras/Raf/MEK/ERK	‑ Promote SC dedifferentiation, proliferation, and demyelination	[71,72]
			‑ Suppress SC differentiation	
			‑ Recruit inflammatory cells	
		‑ Cell-autonomous MAPK/ERK1/2 activation in SCs	‑ Increase SCs myelin sheath thickness	[73]
	(rat)	‑ VEGF-A/VEGFR2/ERK	‑ Promote angiogenesis	[74]
CNS	(frog)	‑ RA/MAPK/ERK&AKT&STAT3	‑ Promote RGC long-term survival	[75]
	(mice)	‑ SORLA/EGFR/ERK/c-fos	‑ Promote neurite outgrowth	[76]
		‑ Bcl-2/Ca^2+^ influx/ERK/CREB	‑ Promote neuronal survival and neurite outgrowth	[24]
		‑ MAPK/ERK1/2 activation	‑ Promote OPC differentiation and remyelination	[77]
		‑ Cell-autonomous MAPK/ERK1/2 activation in OLs/ OPCs	‑ Increase OLs myelin sheath thickness and OPCs proliferation	[73,78]
		‑ EGFR/ERK/decreased TRIM32	‑ Suppress NSC differentiation	[79]
	(rat)	‑ BDNF/trkB/ERK/CREB	‑ Promote neurite outgrowth	[80]
		‑ Intracellular Sigma Peptide/ERK/CREB	‑ Promote axon regrowth and neuron functional recovery	[81]
		‑ ERK/MAPK	‑ Promote axon regrowth	[82]

WE, wound epidermis; EGFR, epidermal growth factor receptor; MMP9, matrix metallopeptidase 9; Akt, protein kinase B; raldh2, retinal dehydrogenase 2; PAP, pilose antler peptide; InsR, insulin receptor; Duox, dual oxidase 2; Nox2, NADPH-oxidase 2; ERBB2, Erb-B2 receptor tyrosine kinase 2; NRG1, neuregulin 1; Dag1, α-dystroglycan; LPA, lysophosphatidic acid; E2F1, E2F transcription factor 1; IGF-1R, insulin-like growth factor type 1 receptor; PKA, protein kinase A; NMII, non-muscle myosin II; HGF, hepatocyte growth factor; GHR, growth hormone receptor; HB-EGF, heparin-binding EGF-like growth factor; *pax6_b_*, paired box 6_b_; PAX6, paired box 6; H3K27me3, tri-methylation of histone H3 at lysine 27; VEGFR2, vascular endothelial growth factor receptor 2; SORLA, sortilin-related receptor with A-type repeats; ISP, intracellular sigma peptide; CREB, the cAMP-response element binding protein; CM, cardiomyocyte; NSC, neural stem cell; RA, retinoic acid; CNS, central nervous system; TRIM32, tripartite motif containing 32.

## Data Availability

Not applicable.

## Abbreviations

FGF2	Fibroblast growth factor 2
IGF-1	Insulin-like growth factor type 1
IGF-1R	Insulin-like growth factor type 1 receptor
BDNF	Brain-derived neurotrophic factor
TGF	Transforming growth factor
ROS	Reactive oxygen species
MEK	Mitogen-activated protein kinase
EGFR	Epidermal growth factor receptor
MMP9	Matrix metallopeptidase 9
FGFR	Fibroblast growth factor receptor
PI3K	Phosphoinositide 3-kinase
Akt	Protein kinase B
IRS-1	Insulin receptor substrate 1
DUSP6	Dual specificity phosphatase 6
YAP	Yes-associated protein
VEGF	Vascular endothelial growth factor
E2F1	E2F transcription factor 1
ECRAR	Endogenous cardiac regeneration-associated regulator
PKA	Protein kinase A
NMII	Non-muscle myosin II
HB-EGF	Heparin-binding EGF-like growth factor
STAT3	Signal transducer and activator of transcription 3
*a* *scl1a*	Achaete-scute complex-like 1a
CREB	The cAMP-response element binding protein
Rb	Retinoblastoma protein
nAG	Newt anterior gradient protein
CM	Cardiomyocyte
ECM	Extracellular matrix
MI	Myocardium infarction
RPE	Retinal pigmented epithelium cell
MG	Müller glia cell
PNS	Peripheral nervous system
CNS	Central nervous system
SC	Schwann cell
OL	Oligodendrocyte
OPC	Oligodendrocyte precursor cell
*Agr*	Anterior gradient

## References

[1] Jazwinska A., Sallin P. (2016). Regeneration versus scarring in vertebrate appendages and heart. J. Pathol..

[2] Tanaka E.M. (2016). The Molecular and Cellular Choreography of Appendage Regeneration. Cell.

[3] Stoick-Cooper C.L., Moon R.T., Weidinger G. (2007). Advances in signaling in vertebrate regeneration as a prelude to regenerative medicine. Genes Dev..

[4] Maienschein J. (2011). Regenerative medicine’s historical roots in regeneration, transplantation, and translation. Dev. Biol..

[5] Goldman J.A., Poss K.D. (2020). Gene regulatory programmes of tissue regeneration. Nat. Rev. Genet..

[6] Busca R., Pouyssegur J., Lenormand P. (2016). ERK1 and ERK2 Map Kinases: Specific Roles or Functional Redundancy?. Front. Cell Dev. Biol..

[7] Tanoue T., Adachi M., Moriguchi T., Nishida E. (2000). A conserved docking motif in MAP kinases common to substrates, activators and regulators. Nat. Cell Biol..

[8] Liu S., Sun J.P., Zhou B., Zhang Z.Y. (2006). Structural basis of docking interactions between ERK2 and MAP kinase phosphatase 3. Proc. Natl. Acad. Sci. USA.

[9] Robbins D.J., Zhen E., Owaki H., Vanderbilt C.A., Ebert D., Geppert T.D., Cobb M.H. (1993). Regulation and properties of extracellular signal-regulated protein kinases 1 and 2 in vitro. J. Biol. Chem..

[10] Lefloch R., Pouyssegur J., Lenormand P. (2009). Total ERK1/2 activity regulates cell proliferation. Cell Cycle.

[11] Park O.J., Kim H.J., Woo K.M., Baek J.H., Ryoo H.M. (2010). FGF2-activated ERK mitogen-activated protein kinase enhances Runx2 acetylation and stabilization. J. Biol. Chem..

[12] Blum Y., Mikelson J., Dobrzynski M., Ryu H., Jacques M.A., Jeon N.L., Khammash M., Pertz O. (2019). Temporal perturbation of ERK dynamics reveals network architecture of FGF2/MAPK signaling. Mol. Syst. Biol..

[13] Lee C.K., Lee H.M., Kim H.J., Park H.J., Won K.J., Roh H.Y., Choi W.S., Jeon B.H., Park T.K., Kim B. (2007). Syk contributes to PDGF-BB-mediated migration of rat aortic smooth muscle cells via MAPK pathways. Cardiovasc. Res..

[14] Zhan Y., Kim S., Izumi Y., Izumiya Y., Nakao T., Miyazaki H., Iwao H. (2003). Role of JNK, p38, and ERK in platelet-derived growth factor-induced vascular proliferation, migration, and gene expression. Arterioscler. Thromb. Vasc. Biol..

[15] Shimizu M., Shirakami Y., Sakai H., Tatebe H., Nakagawa T., Hara Y., Weinstein I.B., Moriwaki H. (2008). EGCG inhibits activation of the insulin-like growth factor (IGF)/IGF-1 receptor axis in human hepatocellular carcinoma cells. Cancer Lett..

[16] Singh A.P., Glennon M.S., Umbarkar P., Gupte M., Galindo C.L., Zhang Q., Force T., Becker J.R., Lal H. (2019). Ponatinib-induced cardiotoxicity: Delineating the signalling mechanisms and potential rescue strategies. Cardiovasc. Res..

[17] Madhu L.N., Kodali M., Attaluri S., Shuai B., Melissari L., Rao X., Shetty A.K. (2021). Melatonin improves brain function in a model of chronic Gulf War Illness with modulation of oxidative stress, NLRP3 inflammasomes, and BDNF-ERK-CREB pathway in the hippocampus. Redox Biol..

[18] Ivanov S.V., Panaccione A., Brown B., Guo Y., Moskaluk C.A., Wick M.J., Brown J.L., Ivanova A.V., Issaeva N., El-Naggar A.K. (2013). TrkC signaling is activated in adenoid cystic carcinoma and requires NT-3 to stimulate invasive behavior. Oncogene.

[19] Watts S.W. (2000). 5-Hydroxytryptamine-induced potentiation of endothelin-1- and norepinephrine-induced contraction is mitogen-activated protein kinase pathway dependent. Hypertension.

[20] Guo G., Gong K., Ali S., Ali N., Shallwani S., Hatanpaa K.J., Pan E., Mickey B., Burma S., Wang D.H. (2017). A TNF-JNK-Axl-ERK signaling axis mediates primary resistance to EGFR inhibition in glioblastoma. Nat. Neurosci..

[21] Eliopoulos A.G., Wang C.C., Dumitru C.D., Tsichlis P.N. (2003). Tpl2 transduces CD40 and TNF signals that activate ERK and regulates IgE induction by CD40. EMBO J..

[22] Appleton C.T., Usmani S.E., Mort J.S., Beier F. (2010). Rho/ROCK and MEK/ERK activation by transforming growth factor-alpha induces articular cartilage degradation. Lab. Investig..

[23] Wan L., Wang Y., Zhang Z., Wang J., Niu M., Wu Y., Yang Y., Dang Y., Hui S., Ni M. (2021). Elevated TEFM expression promotes growth and metastasis through activation of ROS/ERK signaling in hepatocellular carcinoma. Cell Death Dis..

[24] Jiao J., Huang X., Feit-Leithman R.A., Neve R.L., Snider W., Dartt D.A., Chen D.F. (2005). Bcl-2 enhances Ca^2+^ signaling to support the intrinsic regenerative capacity of CNS axons. EMBO J..

[25] Doll M.A., Soltanmohammadi N., Schumacher B. (2019). ALG-2/AGO-Dependent mir-35 Family Regulates DNA Damage-Induced Apoptosis through MPK-1/ERK MAPK Signaling Downstream of the Core Apoptotic Machinery in Caenorhabditis elegans. Genetics.

[26] Ohm A.M., Affandi T., Reyland M.E. (2019). EGF receptor and PKCdelta kinase activate DNA damage-induced pro-survival and pro-apoptotic signaling via biphasic activation of ERK and MSK1 kinases. J. Biol. Chem..

[27] Chang L., Karin M. (2001). Mammalian MAP kinase signalling cascades. Nature.

[28] Maik-Rachline G., Hacohen-Lev-Ran A., Seger R. (2019). Nuclear ERK: Mechanism of Translocation, Substrates, and Role in Cancer. Int. J. Mol. Sci..

[29] Kolch W. (2005). Coordinating ERK/MAPK signalling through scaffolds and inhibitors. Nat. Rev. Mol. Cell Biol..

[30] Kolch W. (2000). Meaningful relationships: The regulation of the Ras/Raf/MEK/ERK pathway by protein interactions. Biochem. J..

[31] Lavoie H., Gagnon J., Therrien M. (2020). ERK signalling: A master regulator of cell behaviour, life and fate. Nat. Rev. Mol. Cell Biol..

[32] Unal E.B., Uhlitz F., Bluthgen N. (2017). A compendium of ERK targets. FEBS Lett..

[33] Yun M.H., Gates P.B., Brockes J.P. (2014). Sustained ERK activation underlies reprogramming in regeneration-competent salamander cells and distinguishes them from their mammalian counterparts. Stem Cell Rep..

[34] Blassberg R.A., Garza-Garcia A., Janmohamed A., Gates P.B., Brockes J.P. (2011). Functional convergence of signalling by GPI-anchored and anchorless forms of a salamander protein implicated in limb regeneration. J. Cell Sci..

[35] Suzuki M., Satoh A., Ide H., Tamura K. (2007). Transgenic Xenopus with prx1 limb enhancer reveals crucial contribution of MEK/ERK and PI3K/AKT pathways in blastema formation during limb regeneration. Dev. Biol..

[36] Franklin B.M., Voss S.R., Osborn J.L. (2017). Ion channel signaling influences cellular proliferation and phagocyte activity during axolotl tail regeneration. Mech. Dev..

[37] Sato K., Umesono Y., Mochii M. (2018). A transgenic reporter under control of an es1 promoter/enhancer marks wound epidermis and apical epithelial cap during tail regeneration in Xenopus laevis tadpole. Dev. Biol..

[38] Yoo S.K., Freisinger C.M., LeBert D.C., Huttenlocher A. (2012). Early redox, Src family kinase, and calcium signaling integrate wound responses and tissue regeneration in zebrafish. J. Cell Biol..

[39] Mathew L.K., Sengupta S., Franzosa J.A., Perry J., La Du J., Andreasen E.A., Tanguay R.L. (2009). Comparative expression profiling reveals an essential role for raldh2 in epimorphic regeneration. J. Biol. Chem..

[40] Owlarn S., Klenner F., Schmidt D., Rabert F., Tomasso A., Reuter H., Mulaw M.A., Moritz S., Gentile L., Weidinger G. (2017). Generic wound signals initiate regeneration in missing-tissue contexts. Nat. Commun..

[41] De Simone A., Evanitsky M.N., Hayden L., Cox B.D., Wang J., Tornini V.A., Ou J., Chao A., Poss K.D., Di Talia S. (2021). Control of osteoblast regeneration by a train of Erk activity waves. Nature.

[42] Kobayashi-Sun J., Suzuki N., Hattori A., Yamaguchi M., Kobayashi I. (2020). Melatonin suppresses both osteoblast and osteoclast differentiation through repression of epidermal Erk signaling in the zebrafish scale. Biochem. Biophys. Res. Commun..

[43] Yun C., Qian W., Wu J., Yuan C., Jiang S., Lv J. (2020). Pilose antler peptide promotes osteoblast proliferation, differentiation and mineralization via the insulin signaling pathway. Exp. Ther. Med..

[44] Li C., Harper A., Puddick J., Wang W., McMahon C. (2012). Proteomes and signalling pathways of antler stem cells. PLoS ONE.

[45] Han P., Zhou X.H., Chang N., Xiao C.L., Yan S., Ren H., Yang X.Z., Zhang M.L., Wu Q., Tang B. (2014). Hydrogen peroxide primes heart regeneration with a derepression mechanism. Cell Res..

[46] Missinato M.A., Saydmohammed M., Zuppo D.A., Rao K.S., Opie G.W., Kuhn B., Tsang M. (2018). Dusp6 attenuates Ras/MAPK signaling to limit zebrafish heart regeneration. Development.

[47] Aharonov A., Shakked A., Umansky K.B., Savidor A., Genzelinakh A., Kain D., Lendengolts D., Revach O.Y., Morikawa Y., Dong J. (2020). ERBB2 drives YAP activation and EMT-like processes during cardiac regeneration. Nat. Cell Biol..

[48] D’Uva G., Aharonov A., Lauriola M., Kain D., Yahalom-Ronen Y., Carvalho S., Weisinger K., Bassat E., Rajchman D., Yifa O. (2015). ERBB2 triggers mammalian heart regeneration by promoting cardiomyocyte dedifferentiation and proliferation. Nat. Cell Biol..

[49] Bassat E., Mutlak Y.E., Genzelinakh A., Shadrin I.Y., Baruch Umansky K., Yifa O., Kain D., Rajchman D., Leach J., Riabov Bassat D. (2017). The extracellular matrix protein agrin promotes heart regeneration in mice. Nature.

[50] Lou X., Zhao M., Fan C., Fast V.G., Valarmathi M.T., Zhu W., Zhang J. (2020). N-cadherin overexpression enhances the reparative potency of human-induced pluripotent stem cell-derived cardiac myocytes in infarcted mouse hearts. Cardiovasc. Res..

[51] Wang F., Liu S., Pei J., Cai L., Liu N., Liang T., Dong X., Cong X., Chun J., Chen J. (2020). LPA(3)-mediated lysophosphatidic acid signaling promotes postnatal heart regeneration in mice. Theranostics.

[52] Strash N., DeLuca S., Janer Carattini G.L., Heo S.C., Gorsuch R., Bursac N. (2021). Human Erbb2-induced Erk activity robustly stimulates cycling and functional remodeling of rat and human cardiomyocytes. eLife.

[53] Chen Y., Li X., Li B., Wang H., Li M., Huang S., Sun Y., Chen G., Si X., Huang C. (2019). Long Non-coding RNA ECRAR Triggers Post-natal Myocardial Regeneration by Activating ERK1/2 Signaling. Mol. Ther..

[54] Ohashi A., Saito N., Kashimoto R., Furukawa S., Yamamoto S., Satoh A. (2020). Axolotl liver regeneration is accomplished via compensatory congestion mechanisms regulated by ERK signaling after partial hepatectomy. Dev. Dyn..

[55] Fang Y., Liu C., Shu B., Zhai M., Deng C., He C., Luo M., Han T., Zheng W., Zhang J. (2018). Axis of serotonin-pERK-YAP in liver regeneration. Life Sci..

[56] Desbois-Mouthon C., Wendum D., Cadoret A., Rey C., Leneuve P., Blaise A., Housset C., Tronche F., Le Bouc Y., Holzenberger M. (2006). Hepatocyte proliferation during liver regeneration is impaired in mice with liver-specific IGF-1R knockout. FASEB J..

[57] Abu Rmilah A.A., Zhou W., Nyberg S.L. (2020). Hormonal Contribution to Liver Regeneration. Mayo Clin. Proc. Innov. Qual. Outcomes.

[58] Wang X., Ni C., Jiang N., Wei J., Liang J., Zhao B., Lin X. (2020). Generation of liver bipotential organoids with a small-molecule cocktail. J. Mol. Cell. Biol..

[59] Kim Y., Kang K., Lee S.B., Seo D., Yoon S., Kim S.J., Jang K., Jung Y.K., Lee K.G., Factor V.M. (2019). Small molecule-mediated reprogramming of human hepatocytes into bipotent progenitor cells. J. Hepatol..

[60] Zerrad-Saadi A., Lambert-Blot M., Mitchell C., Bretes H., Collin de l’Hortet A., Baud V., Chereau F., Sotiropoulos A., Kopchick J.J., Liao L. (2011). GH receptor plays a major role in liver regeneration through the control of EGFR and ERK1/2 activation. Endocrinology.

[61] Svegliati-Baroni G., Ridolfi F., Caradonna Z., Alvaro D., Marzioni M., Saccomanno S., Candelaresi C., Trozzi L., Macarri G., Benedetti A. (2003). Regulation of ERK/JNK/p70S6K in two rat models of liver injury and fibrosis. J. Hepatol..

[62] Wan J., Ramachandran R., Goldman D. (2012). HB-EGF is Necessary and Sufficient for Muller Glia Dedifferentiation and Retina Regeneration. Dev. Cell.

[63] Wan J., Zhao X.F., Vojtek A., Goldman D. (2014). Retinal Injury, Growth Factors, and Cytokines Converge on beta-Catenin and pStat3 Signaling to Stimulate Retina Regeneration. Cell Rep..

[64] Mizuno A., Yasumuro H., Yoshikawa T., Inami W., Chiba C. (2012). MEK-ERK signaling in adult newt retinal pigment epithelium cells is strengthened immediately after surgical induction of retinal regeneration. Neurosci. Lett..

[65] Yasumuro H., Sakurai K., Toyama F., Maruo F., Chiba C. (2017). Implications of a Multi-Step Trigger of Retinal Regeneration in the Adult Newt. Biomedicines.

[66] Yoshikawa T., Mizuno A., Yasumuro H., Inami W., Vergara M.N., Del Rio-Tsonis K., Chiba C. (2012). MEK-ERK and heparin-susceptible signaling pathways are involved in cell-cycle entry of the wound edge retinal pigment epithelium cells in the adult newt. Pigment Cell Melanoma Res..

[67] Susaki K., Chiba C. (2007). MEK mediates in vitro neural transdifferentiation of the adult newt retinal pigment epithelium cells: Is FGF2 an induction factor?. Pigment Cell Res..

[68] Vergara M.N., Del Rio-Tsonis K. (2009). Retinal regeneration in the Xenopus laevis tadpole: A new model system. Mol. Vis..

[69] Spence J.R., Madhavan M., Aycinena J.C., Del Rio-Tsonis K. (2007). Retina regeneration in the chick embryo is not induced by spontaneous Mitf downregulation but requires FGF/FGFR/MEK/Erk dependent upregulation of Pax6. Mol. Vis..

[70] Bao B., He Y., Tang D., Li W., Li H. (2017). Inhibition of H3K27me3 Histone Demethylase Activity Prevents the Proliferative Regeneration of Zebrafish Lateral Line Neuromasts. Front. Mol. Neurosci..

[71] Harrisingh M.C., Perez-Nadales E., Parkinson D.B., Malcolm D.S., Mudge A.W., Lloyd A.C. (2004). The Ras/Raf/ERK signalling pathway drives Schwann cell dedifferentiation. EMBO J..

[72] Napoli I., Noon L.A., Ribeiro S., Kerai A.P., Parrinello S., Rosenberg L.H., Collins M.J., Harrisingh M.C., White I.J., Woodhoo A. (2012). A central role for the ERK-signaling pathway in controlling Schwann cell plasticity and peripheral nerve regeneration in vivo. Neuron.

[73] Ishii A., Furusho M., Bansal R. (2013). Sustained activation of ERK1/2 MAPK in oligodendrocytes and schwann cells enhances myelin growth and stimulates oligodendrocyte progenitor expansion. J. Neurosci..

[74] Wu P., Tong Z., Luo L., Zhao Y., Chen F., Li Y., Huselstein C., Ye Q., Ye Q., Chen Y. (2021). Comprehensive strategy of conduit guidance combined with VEGF producing Schwann cells accelerates peripheral nerve repair. Bioact. Mater..

[75] Duprey-Diaz M.V., Blagburn J.M., Blanco R.E. (2016). Exogenous Modulation of Retinoic Acid Signaling Affects Adult RGC Survival in the Frog Visual System after Optic Nerve Injury. PLoS ONE.

[76] Stupack J., Xiong X.P., Jiang L.L., Zhang T., Zhou L., Campos A., Ranscht B., Mobley W., Pasquale E.B., Xu H. (2020). Soluble SORLA Enhances Neurite Outgrowth and Regeneration through Activation of the EGF Receptor/ERK Signaling Axis. J. Neurosci..

[77] Najm F.J., Madhavan M., Zaremba A., Shick E., Karl R.T., Factor D.C., Miller T.E., Nevin Z.S., Kantor C., Sargent A. (2015). Drug-based modulation of endogenous stem cells promotes functional remyelination in vivo. Nature.

[78] Ishii A., Fyffe-Maricich S.L., Furusho M., Miller R.H., Bansal R. (2012). ERK1/ERK2 MAPK signaling is required to increase myelin thickness independent of oligodendrocyte differentiation and initiation of myelination. J. Neurosci..

[79] Xue W., Zhao Y., Xiao Z., Wu X., Ma D., Han J., Li X., Xue X., Yang Y., Fang Y. (2020). Epidermal growth factor receptor-extracellular-regulated kinase blockade upregulates TRIM32 signaling cascade and promotes neurogenesis after spinal cord injury. Stem Cells.

[80] Hollis E.R., Jamshidi P., Low K., Blesch A., Tuszynski M.H. (2009). Induction of corticospinal regeneration by lentiviral trkB-induced Erk activation. Proc. Natl. Acad. Sci. USA.

[81] Yao M., Sun H., Yuan Q., Li N., Li H., Tang Y., Leung G.K., Wu W. (2019). Targeting proteoglycan receptor PTPsigma restores sensory function after spinal cord dorsal root injury by activation of Erks/CREB signaling pathway. Neuropharmacology.

[82] Huang H., Liu H., Yan R., Hu M. (2017). PI3K/Akt and ERK/MAPK Signaling Promote Different Aspects of Neuron Survival and Axonal Regrowth Following Rat Facial Nerve Axotomy. Neurochem. Res..

[83] Morgan T.H. (1901). Regeneration and Liability to Injury. Science.

[84] Lin T.Y., Gerber T., Taniguchi-Sugiura Y., Murawala P., Hermann S., Grosser L., Shibata E., Treutlein B., Tanaka E.M. (2021). Fibroblast dedifferentiation as a determinant of successful regeneration. Dev. Cell.

[85] Kragl M., Knapp D., Nacu E., Khattak S., Maden M., Epperlein H.H., Tanaka E.M. (2009). Cells keep a memory of their tissue origin during axolotl limb regeneration. Nature.

[86] Tanaka H.V., Ng N.C.Y., Yang Yu Z., Casco-Robles M.M., Maruo F., Tsonis P.A., Chiba C. (2016). A developmentally regulated switch from stem cells to dedifferentiation for limb muscle regeneration in newts. Nat. Commun..

[87] Niethammer P. (2016). The early wound signals. Curr. Opin. Genet. Dev..

[88] Love N.R., Chen Y., Ishibashi S., Kritsiligkou P., Lea R., Koh Y., Gallop J.L., Dorey K., Amaya E. (2013). Amputation-induced reactive oxygen species are required for successful Xenopus tadpole tail regeneration. Nat. Cell Biol..

[89] Ferreira F., Raghunathan V., Luxardi G., Zhu K., Zhao M. (2018). Early redox activities modulate Xenopus tail regeneration. Nat. Commun..

[90] Tu M.K., Borodinsky L.N. (2014). Spontaneous calcium transients manifest in the regenerating muscle and are necessary for skeletal muscle replenishment. Cell Calcium.

[91] Kujawski S., Lin W., Kitte F., Börmel M., Fuchs S., Arulmozhivarman G., Vogt S., Theil D., Zhang Y., Antos C.L. (2014). Calcineurin regulates coordinated outgrowth of zebrafish regenerating fins. Dev. Cell.

[92] Ozkucur N., Epperlein H.H., Funk R.H. (2010). Ion imaging during axolotl tail regeneration in vivo. Dev. Dyn..

[93] Okuda K.S., Keyser M.S., Gurevich D.B., Sturtzel C., Mason E.A., Paterson S., Chen H., Scott M., Condon N.D., Martin P. (2021). Live-imaging of endothelial Erk activity reveals dynamic and sequential signalling events during regenerative angiogenesis. eLlife.

[94] Wang H., Lööf S., Borg P., Nader G.A., Blau H.M., Simon A. (2015). Turning terminally differentiated skeletal muscle cells into regenerative progenitors. Nat. Commun..

[95] Yun M.H., Gates P.B., Brockes J.P. (2013). Regulation of p53 is critical for vertebrate limb regeneration. Proc. Natl. Acad. Sci. USA.

[96] Saera-Vila A., Kish P.E., Kahana A. (2016). Fgf regulates dedifferentiation during skeletal muscle regeneration in adult zebrafish. Cell. Signal..

[97] Hino N., Rossetti L., Marin-Llaurado A., Aoki K., Trepat X., Matsuda M., Hirashima T. (2020). ERK-Mediated Mechanochemical Waves Direct Collective Cell Polarization. Dev. Cell.

[98] Kierdorf U., Li C., Price J.S. (2009). Improbable appendages: Deer antler renewal as a unique case of mammalian regeneration. Semin. Cell. Dev. Biol..

[99] Korotkova D.D., Lyubetsky V.A., Ivanova A.S., Rubanov L.I., Seliverstov A.V., Zverkov O.A., Martynova N.Y., Nesterenko A.M., Tereshina M.B., Peshkin L. (2019). Bioinformatics Screening of Genes Specific for Well-Regenerating Vertebrates Reveals c-answer, a Regulator of Brain Development and Regeneration. Cell Rep..

[100] Kumar A., Godwin J.W., Gates P.B., Garza-Garcia A.A., Brockes J.P. (2007). Molecular basis for the nerve dependence of limb regeneration in an adult vertebrate. Science.

[101] Grassme K.S., Garza-Garcia A., Delgado J.P., Godwin J.W., Kumar A., Gates P.B., Driscoll P.C., Brockes J.P. (2016). Mechanism of Action of Secreted Newt Anterior Gradient Protein. PLoS ONE.

[102] Satoh A., Graham G.M., Bryant S.V., Gardiner D.M. (2008). Neurotrophic regulation of epidermal dedifferentiation during wound healing and limb regeneration in the axolotl (*Ambystoma mexicanum*). Dev. Biol..

[103] Vinarsky V., Atkinson D.L., Stevenson T.J., Keating M.T., Odelberg S.J. (2005). Normal newt limb regeneration requires matrix metalloproteinase function. Dev. Biol..

[104] Jopling C., Sleep E., Raya M., Marti M., Raya A., Izpisua Belmonte J.C. (2010). Zebrafish heart regeneration occurs by cardiomyocyte dedifferentiation and proliferation. Nature.

[105] Gonzalez-Rosa J.M., Peralta M., Mercader N. (2012). Pan-epicardial lineage tracing reveals that epicardium derived cells give rise to myofibroblasts and perivascular cells during zebrafish heart regeneration. Dev. Biol..

[106] Kikuchi K., Gupta V., Wang J., Holdway J.E., Wills A.A., Fang Y., Poss K.D. (2011). *tcf21^+^* epicardial cells adopt non-myocardial fates during zebrafish heart development and regeneration. Development.

[107] Mercer S.E., Odelberg S.J., Simon H.G. (2013). A dynamic spatiotemporal extracellular matrix facilitates epicardial-mediated vertebrate heart regeneration. Dev. Biol..

[108] Porrello E.R., Mahmoud A.I., Simpson E., Hill J.A., Richardson J.A., Olson E.N., Sadek H.A. (2011). Transient regenerative potential of the neonatal mouse heart. Science.

[109] Vivien C.J., Hudson J.E., Porrello E.R. (2016). Evolution, comparative biology and ontogeny of vertebrate heart regeneration. NPJ Regen. Med..

[110] Uygur A., Lee R.T. (2016). Mechanisms of Cardiac Regeneration. Dev. Cell.

[111] Liu P., Zhong T.P. (2017). MAPK/ERK signalling is required for zebrafish cardiac regeneration. Biotechnol. Lett..

[112] Miyajima A., Tanaka M., Itoh T. (2014). Stem/progenitor cells in liver development, homeostasis, regeneration, and reprogramming. Cell Stem Cell.

[113] Preziosi M.E., Monga S.P. (2017). Update on the Mechanisms of Liver Regeneration. Semin. Liver Dis..

[114] Fausto N. (2004). Liver regeneration and repair: Hepatocytes, progenitor cells, and stem cells. Hepatology.

[115] Michalopoulos G.K., Bhushan B. (2021). Liver regeneration: Biological and pathological mechanisms and implications. Nat. Rev. Gastroenterol. Hepatol..

[116] Borowiak M., Garratt A.N., Wustefeld T., Strehle M., Trautwein C., Birchmeier C. (2004). Met provides essential signals for liver regeneration. Proc. Natl. Acad. Sci. USA.

[117] Araujo T.G., de Oliveira A.G., Tobar N., Saad M.J., Moreira L.R., Reis E.R., Nicola E.M., de Jorge G.L., dos Tartaro R.R., Boin I.F. (2013). Liver regeneration following partial hepatectomy is improved by enhancing the HGF/Met axis and Akt and Erk pathways after low-power laser irradiation in rats. Lasers Med. Sci..

[118] Wilken M.S., Reh T.A. (2016). Retinal regeneration in birds and mice. Curr. Opin. Genet. Dev..

[119] Yoshii C., Ueda Y., Okamoto M., Araki M. (2007). Neural retinal regeneration in the anuran amphibian Xenopus laevis post-metamorphosis: Transdifferentiation of retinal pigmented epithelium regenerates the neural retina. Dev. Biol..

[120] Goldman D. (2014). Muller glial cell reprogramming and retina regeneration. Nat. Rev. Neurosci..

[121] Langhe R., Chesneau A., Colozza G., Hidalgo M., Ail D., Locker M., Perron M. (2017). Müller glial cell reactivation in Xenopus models of retinal degeneration. Glia.

[122] Powell C., Grant A.R., Cornblath E., Goldman D. (2013). Analysis of DNA methylation reveals a partial reprogramming of the Muller glia genome during retina regeneration. Proc. Natl. Acad. Sci. USA.

[123] Kha C.X., Guerin D.J., Tseng K.A. (2019). Using the Xenopus Developmental Eye Regrowth System to Distinguish the Role of Developmental Versus Regenerative Mechanisms. Front. Physiol..

[124] Catala M., Kubis N. (2013). Gross anatomy and development of the peripheral nervous system. Handb. Clin. Neurol..

[125] Rigoni M., Negro S. (2020). Signals Orchestrating Peripheral Nerve Repair. Cells.

[126] Min Q., Parkinson D.B., Dun X.P. (2021). Migrating Schwann cells direct axon regeneration within the peripheral nerve bridge. Glia.

[127] Nocera G., Jacob C. (2020). Mechanisms of Schwann cell plasticity involved in peripheral nerve repair after injury. Cell. Mol. Life Sci..

[128] Negro S., Bergamin E., Rodella U., Duregotti E., Scorzeto M., Jalink K., Montecucco C., Rigoni M. (2016). ATP Released by Injured Neurons Activates Schwann Cells. Front. Cell Neurosci..

[129] Cervellini I., Galino J., Zhu N., Allen S., Birchmeier C., Bennett D.L. (2018). Sustained MAPK/ERK Activation in Adult Schwann Cells Impairs Nerve Repair. J. Neurosci..

[130] Cruz I.A., Kappedal R., Mackenzie S.M., Hailey D.W., Hoffman T.L., Schilling T.F., Raible D.W. (2015). Robust regeneration of adult zebrafish lateral line hair cells reflects continued precursor pool maintenance. Dev. Biol..

[131] Denans N., Baek S., Piotrowski T. (2019). Comparing Sensory Organs to Define the Path for Hair Cell Regeneration. Annu. Rev. Cell Dev. Biol..

[132] Curcio M., Bradke F. (2018). Axon Regeneration in the Central Nervous System: Facing the Challenges from the Inside. Annu. Rev. Cell Dev. Biol..

[133] Diaz Quiroz J.F., Echeverri K. (2013). Spinal cord regeneration: Where fish, frogs and salamanders lead the way, can we follow?. Biochem. J..

[134] Munoz R., Edwards-Faret G., Moreno M., Zuniga N., Cline H., Larrain J. (2015). Regeneration of *Xenopus laevis* spinal cord requires Sox2/3 expressing cells. Dev. Biol..

[135] Romero-Alemán M.M., Monzón-Mayor M., Yanes C., Lang D. (2004). Radial glial cells, proliferating periventricular cells, and microglia might contribute to successful structural repair in the cerebral cortex of the lizard Gallotia galloti. Exp. Neurol..

[136] Zukor K.A., Kent D.T., Odelberg S.J. (2011). Meningeal cells and glia establish a permissive environment for axon regeneration after spinal cord injury in newts. Neural Dev..

[137] Joven A., Simon A. (2018). Homeostatic and regenerative neurogenesis in salamanders. Prog. Neurobiol..

[138] Suo N., Guo Y.E., He B., Gu H., Xie X. (2019). Inhibition of MAPK/ERK pathway promotes oligodendrocytes generation and recovery of demyelinating diseases. Glia.

[139] Keyes J., Ganesan A., Molinar-Inglis O., Hamidzadeh A., Zhang J., Ling M., Trejo J., Levchenko A., Zhang J. (2020). Signaling diversity enabled by Rap1-regulated plasma membrane ERK with distinct temporal dynamics. eLife.

[140] Ivanova A.S., Tereshina M.B., Ermakova G.V., Belousov V.V., Zaraisky A.G. (2013). Agr genes, missing in amniotes, are involved in the body appendages regeneration in frog tadpoles. Sci. Rep..

[141] Ivanova A.S., Korotkova D.D., Ermakova G.V., Martynova N.Y., Zaraisky A.G., Tereshina M.B. (2018). Ras-dva small GTPases lost during evolution of amniotes regulate regeneration in anamniotes. Sci. Rep..

[142] Gerber T., Murawala P., Knapp D., Masselink W., Schuez M., Hermann S., Gac-Santel M., Nowoshilow S., Kageyama J., Khattak S. (2018). Single-cell analysis uncovers convergence of cell identities during axolotl limb regeneration. Science.

[143] Aztekin C., Hiscock T.W., Marioni J.C., Gurdon J.B., Simons B.D., Jullien J. (2019). Identification of a regeneration-organizing cell in the Xenopus tail. Science.

[144] Sanor L.D., Flowers G.P., Crews C.M. (2020). Multiplex CRISPR/Cas screen in regenerating haploid limbs of chimeric Axolotls. eLife.

[145] Wang W., Hu C.K., Zeng A., Alegre D., Hu D., Gotting K., Ortega Granillo A., Wang Y., Robb S., Schnittker R. (2020). Changes in regeneration-responsive enhancers shape regenerative capacities in vertebrates. Science.

